# Genomic characteristics of the multiresistant *Acinetobacter baumannii* global clone 1 reference strain A297/RUH875

**DOI:** 10.1093/jac/dkaf160

**Published:** 2025-05-19

**Authors:** Jonathan Koong, Ruth M Hall, Mehrad Hamidian

**Affiliations:** Australian Institute for Microbiology and Infection, University of Technology Sydney, Ultimo, NSW 2007, Australia; School of Life and Environmental Sciences, The University of Sydney, NSW 2006, Australia; Australian Institute for Microbiology and Infection, University of Technology Sydney, Ultimo, NSW 2007, Australia

## Abstract

**Objectives:**

*Acinetobacter baumannii* ST1, also known as global clone 1 (GC1), is a globally distributed lineage associated with antimicrobial resistance (AMR). The multiresistant isolate A297/RUH8751, recovered from a urinary tract infection in the Netherlands in 1984, has served as a reference strain for ST1. We aimed to generate and analyse the complete genome sequence of A297/RUH8751 to provide insights into its genomic features, antibiotic resistance determinants and phylogenetic placement.

**Methods:**

WGS was performed using Oxford Nanopore GridION and Illumina HiSeq platforms. Assembly was conducted with Autocycler v0.2.1. Genomic features, including antibiotic resistance genes, insertion sequences and restriction-modification systems, were characterized using ResFinder, ISFinder and REBASE. Comparative analyses were conducted with the draft genome of NIPH 527, which also represents RUH875, to assess sequence variations. Phylogenetic analysis was performed to determine the evolutionary placement of A297/RUH875 within GC1.

**Results:**

The complete genome of RUH875 (A297) consists of a 3 965 450 bp chromosome and three plasmids: pA297-1 (pRAY*; 6078 bp), pA297-2 (8731 bp) and pA297-3 (a 200 kb conjugative plasmid). The chromosome harbours the AbaR21 genomic resistance island, carrying seven antibiotic resistance genes and six prophage regions. Notably, *bap1* and *bap2*, biofilm-associated genes, were fully resolved, with *bap1* identical to that of *A. baumannii* A1—the earliest GC1 isolate. Comparative analysis with A1 revealed 122 SNPs, with clustered variations suggesting potential recombination events. Phylogenetic analysis confirmed A297/RUH875 as a distinct lineage within GC1. The completed genome sequence provides a reference, in addition to strain A1, for studying AMR evolution in GC1 and enhances comparative genomic analyses.

## Introduction

MDR, XDR and pan-resistant isolates of *Acinetobacter baumannii* are predominantly members of two globally distributed STs: ST1 (Institut Pasteur); and ST2 (also known as global clones 1 and 2 or simply GC1 and GC2, respectively).^1,2^ Accessing genome sequence data of historical and reference strains is essential for tracking the genetic evolution of antibiotic resistance and understanding the mechanisms behind the spread of these resistant isolates. The multiresistant isolate RUH875^3^ was recovered in Dordrecht, The Netherlands from a urinary tract infection in 1984. It has been used in many studies as a reference strain for ST1 (GC1) and ST1 isolates are now known to be distributed globally as they have been found in Asia, Australia,^4^ the Middle East region,^5^ Europe, the USA, Africa^6,7^ and in South America.^8^

RUH875 was stored as A297^9^ in the laboratory of Dr Kevin Towner at the Nottingham University Hospitals NHS Trust, UK and sent to the Hall laboratory where it was stored at −80˚C. We previously reported the draft genome of A297^4^ (GenBank no. FBWR00000000) and also showed it contains three plasmids, namely pA297-1 (6078 bp; GenBank no. KU869529.1), which is also known as pRAY*,^10^ pA297-2 (8731 bp; GenBank no. KU869528.1) encoding an R3-T1 replication protein, and pA297-3 (200 633 bp conjugative plasmid with an MPF_I_ transfer system; GenBank no. KU744946.1).^11^ In addition, pA297-3 has been shown to mobilize pRAY* at high frequency.^12^ Here, we report the complete genome sequence of the multiresistant *A. baumannii* isolate A297 representing RUH875 and compare it with the draft genome of NIPH 527,^13^ which also represents RUH857 that was stored in the laboratory of Dr Alexandre Nemec in the Czech Republic.

## Materials and methods

### WGS and assembly

Whole-cell DNA was extracted from A297 cells grown at 37°C in LB inoculated from a single colony as described previously.^4^ The DNA was prepared, barcoded and long-read sequenced at the Ramaciotti Centre for Genomics (UNSW Sydney, Australia) using a GridION GXB03456 (Oxford Nanopore Technology), flow cell FLO-MIN114 and kit SQKRBK114-96. Sequencing was run using the high-accuracy model basecalling (v4.3.0, 400 bp) and a minimum Q score of 9. The GridION reads were quality checked using Filtlong v0.2.1 (https://github.com/rrwick/Filtlong) with their adaptors removed using Porechop v0.2.4 (https://github.com/rrwick/Porechop) using default parameters. Short-read Illumina HiSeq data, determined previously^4^ with 100 ×  coverage and N50: 110 bp (SRA accession number ERS192746), were quality checked using the FastQC program (https://www.bioinformatics.babraham.ac.uk/projects/fastqc/) followed by trimming adaptors using the Trimmomatic program.^14^ The GridION reads were combined with Illumina HiSeq using Autocycler v0.2.1 (https://github.com/rrwick/Autocycler) to generate a complete assembly.

### Sequence and phylogenetic analysis

Antibiotic resistance genes, insertion sequences and surface polysaccharides that were previously reported by Holt *et al.* in 2016,^4^ were confirmed using the ResFinder,^15^ ISFinder^16^ and Kaptive^17^ programs. REBASE was used to identify restriction/modification genes (https://rebase.neb.com/rebase/rebase.html). CRISPR-Cas types were found using the CRISPR-CasFinder database (https://crisprcas.i2bc.paris-saclay.fr/CrisprCasFinder/Index). Proksee software was used to align the genomes of A297, NIPH527 and A1, and to generate the circular alignment map.^18^ PHASTEST was used to identify prophage regions in A297, NIPH527 and A1.^19^

### Phylogenetic analysis

For comparative analyses, eight additional GC1 genomes representing the main clades and lineages within GC1 (as previously determined by Koong *et al.*^20^) were included in this study. Whole-genome alignments were produced using Panaroo v1.3.2 (https://github.com/gtonkinhill/panaroo). SNPs acquired by recombination were filtered using Gubbins v.2.4.1^21^ and fed into IQ-Tree2 v2.2.0.3 (http://www.iqtree.org/) to produce a maximum-likelihood phylogenetic tree. The final maximum-likelihood phylogenetic tree was inferred from the resulting alignment using RAxML v8 (https://cme.h-its.org/exelixis/web/software/raxml/index.html) with the generalized time-reversible (GTR) gamma model of nucleotide substitution, as previously described. The phylogenetic tree was visualized using the ggtree package^22^ in R.

## Figures and Tables

**Figure 1.**
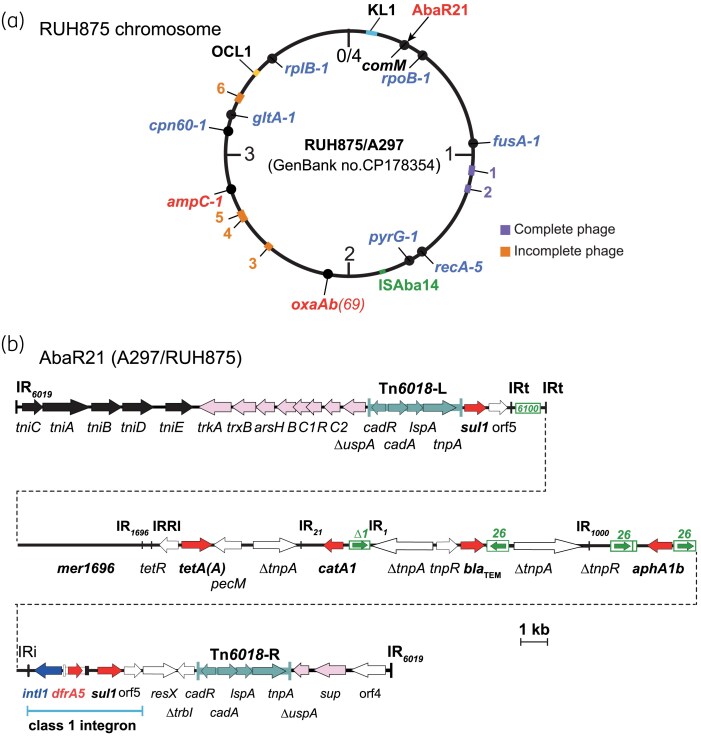
Circular map of the chromosome of RUH875/A (A297) (a) and structure of the AbaR21 genomic resistance island (b). (a) Represents a schematic circular map of the RUH875(A297) chromosome with the position of AbaR21, *ampC*, K and OC, and Institut Pasteur MLST loci marked. Chromosomal locations numbered 1–6 represent phage genomes. Dark purple indicates complete phage genomes and orange shows incomplete phage regions. In (b), the thick central line shows the AbaR21 genomic resistance island, with horizontal arrows indicating the extent and orientation of genes. Antibiotic resistance genes are shown using red arrows. Two terminal vertical lines indicate the inverted repeats (IRs) of AbaR21. Tn*6018* copies are shown using dark green.

**Figure 2.**
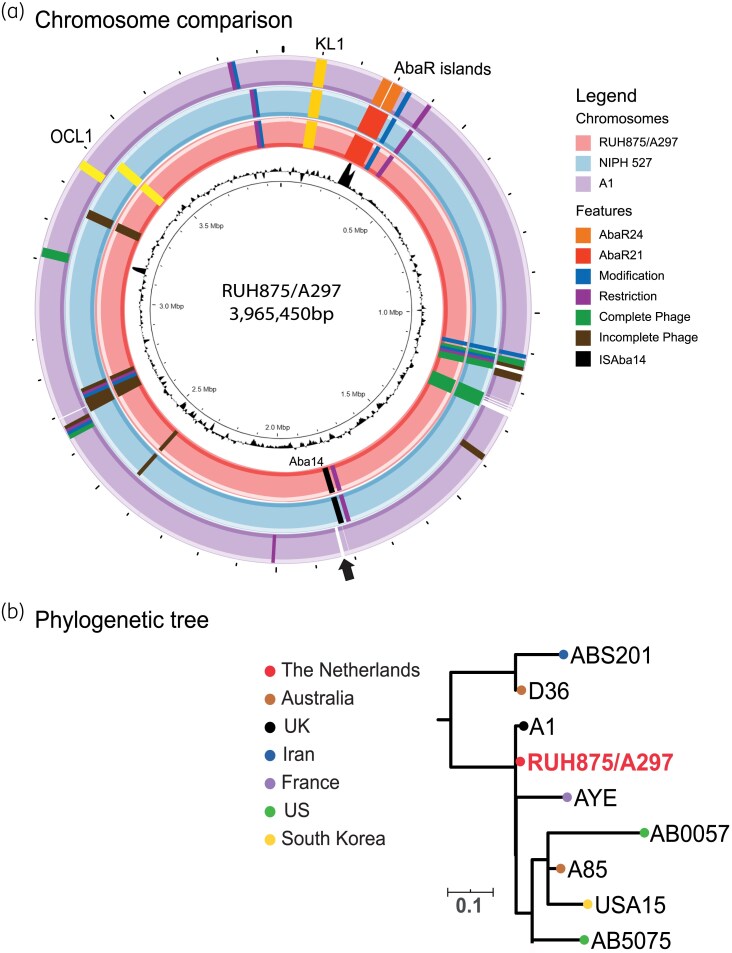
Comparison of the RUH875/A297, NIPU527 and A1 chromosomes. In (a), coloured rings represent BLAST searches comparing chromosomes, with white spaces indicating gaps in the sequence alignment. Solid blocks on the rings represent features located in each chromosome and their approximate location and their names, listed in the legend. The innermost ring indicates the GC content (drawn based on the A297/RUH875 chromosome). The alignment and circular map was created using Proksee software (https://proksee.ca/). (b) The phylogenetic relationship of A297/RUH875 compared with well-known GC1 strains, including the earliest GC1 strain available (A1), is shown.

## Data Availability

The A297/RUH875 complete genome has been deposited in the GenBank/EMBL/DDBJ databases and is publicly available under the following BioProject PRJNA224116 and genome accession numbers: CP178354 (chromosome); CP178355 (pA297-1); CP178356 (pA297-2); and CP178357 (pA297-3).

## Results and discussion

The complete genome of A297/RUH875 consists of the chromosome (3 965 450 bp; GenBank no. CP178354) and three plasmids, pA297-1 (GenBank no. CP178355), pA297-2 (GenBank no. CP178356) and pA297-3 (GenBank no. CP178357). pA297-1 and pA297-3 sequences were identical to those we previously determined using Illumina reads combined with Sanger sequencing of overlapping PCR products. However, pA297-2 differed by two SNPs compared with GenBank KU869528.1. The complete genome confirms our previous report^4^ that A297/RUH875 belongs to ST1 (Institut Pasteur) and to ST231 (Oxford scheme) and carries the KL1 and OCL1 surface polysaccharides, making A297/RUH875 an ST1:ST203:KL1:OCL1 strain.^4^ The completed chromosome also allowed the two large genes encoding biofilm-associated proteins *bap1* (10 089 bp) and *bap2* (25 347 bp) to be completed. Notably, *bap1* is identical to that found in *A. baumannii* strain A1 (GenBank no. CP010781), which was isolated in 1982 in the UK and is the earliest GC1 isolate available.^23^ However, *bap2* is 516 bp shorter than in A1 (due to the absence of a 516 bp sequence repeat unit) and but is otherwise 99.8% identical (54 SNPs and 2 indels in 25 347 bp). The RUH875/A297 chromosome contains five genes that encode type II restriction modification functions including ACKWDP_01585, _5400 and _15820 (encoding methylases) and ACKWDP_18885 (encoding restriction and methylation functions). A297/RUH875 and NIPH 527 contain the same CRISPR-Cas type (with the IF CAS-type); however, A297/RUH875 contained 53 spacers (TTTCTAAATGGCGTATGCCGCCATGAAC) while NIPH 527 only included 26. The RUH875/A297 chromosome also contains six prophage regions (numbered 1–6 in Figure 1a) including four complete and two incomplete prophage genomes. Notably, in addition to all six phage regions, NIPH 527 included a partial phage region (bases 1320–22 954 in contig 10; GenBank no. APQW01000010.1), which was not present in A297/RUH875. Previous studies have documented genomic alterations in *A. baumannii* strains such as ATCC 17978 and ATCC 19606, presumably as a result of long-term storage in different laboratories.^24,25^ Our findings similarly suggest that varying storage conditions—and potentially differing environmental exposures— are likely to have led to genomic changes in A297/RUH875 and NIPH 527.

Here, we found the structure of the AbaR21 genomic resistance island (Figure 1b)^26^ differed only by three SNPs (scattered throughout the island) from the sequence we determined previously using Illumina short reads and sequencing overlapping PCR products (GenBank no. KM921776). AbaR21, found in *comM* in the chromosome, is a derivative of the AbaR0-type island and contains seven antibiotic resistance genes including *sul1* (two copies), *tet*(A), *catA1*, *bla*_TEM_, *aphA1b* and *dfrA5*. In the class 1 integron within AbaR21, the *dfrA5* gene cassette replaces the *aacC1*-ofrP-orfP-ofrQ-*aadA1* array, which is more commonly found in AbaR-type islands.^27^ To the best of our knowledge, AbaR21 is the only AbaR island with the *dfrA5* gene cassette described to date, making it a non-ideal genome for comparative analysis of AbaR-type genomic resistance islands. We recommend the A1 genome (GenBank no. CP010781)^23^ for such analysis as it contains a more standard AbaR-type island structure. The A297 genome also includes the *strAB* streptomycin/spectinomycin resistance and *sul2* sulphonamide resistance genes, which are located on the 200 kb conjugative plasmid pA297-3, as well as the *aadB* tobramycin, gentamicin and kanamycin resistance gene, carried by the small mobilizable plasmid pA297-1 (also known as pRAY*), which can be mobilized by pA297-3.^12^

*A. baumannii* chromosomes often contain several insertion sequences (ISs), in particular ISAba1. Here, in addition to three IS*26* copies and an IS*6100* located in AbaR21, we only found a single ISAba14 at bases 1 810 143–1 811 424. Notably, no ISAba1 were found in the A297 chromosome (Figure 1a), although there is one copy of ISAba1 in pA297-3.

We used the sequences of the A297 plasmids (pA297-1, pA297-2 and pA297-3) to identify several contigs that could reconstitute all three complete plasmids in the NIPH 527 draft genome (GenBank no. APQW01000000), which also represents RUH875.^13^ The genome sequence of A279/RUH875 differs from NIPH 527 by only 17 bp, confirming that both represent the same strains. However, comparing the complete genome of A279/RUH875 to the complete chromosome of A1 also revealed that all six phage regions except number two (Figure 2a) were also present in the A1 genome. An 8922 bp segment (bases 1 821 084–1 836 337 in the A297/RUH875 chromosome; GenBank accession CP178354) is absent from the A1 chromosome (shown by an arrow in Figure 2a). This region encodes several hypothetical proteins, as well as a few phage-associated proteins (e.g. the major capsid protein ACKWDP_08995). However, PHASTEST did not identify this segment as a potential prophage region. Notably, this segment is present in the majority of complete *A. baumannii* genomes. Moreover, the chromosomes differ by 122 SNPs and almost half of these (*n* = 54) were clustered in three chromosomal regions: (i) 21 SNPs in a 397 bp region between bases 1 128 148 and 1 128  544; (ii) 15 SNPs in a 238 bp region between bases 1 132 064 and 1 132 301; and (iii) 18 SNPs between 1 142 070 and 1 142 462 in a 393 bp region. We found sequences identical to these regions from A297/RUH875 in only two GC1 genomes, AYE (CU459141.1; recovered in 2006 in France^28^) and AB169-VUB (CP091367.1; recovered prior to 2022 in Belgium). They were also present in two strains belonging to ST32 [AR_0070 (GenBank no. CP027178.1) and AR_0052 (GenBank no. CP027183.1)]. The remaining SNPs were scattered throughout the chromosome. This suggests that potential early recombination events led to the divergence of A297/RUH857 (from its more ancestral strain, e.g. A1; CP010781). The three recombinant regions in A297/RUH857 were identical to the corresponding chromosomal segments in NIPH 527. Phylogenetic analysis with representative GC1 isolates showed that A297/RUH857 belongs to a distinct branch, in the main clade (lineage 1), indicating that it represents a novel lineage (Figure 2b; see also Figure 2 of the report by Koong *et al.*^20^ in 2021 and Figure 1 of the report by Holt *et al.*^4^ in 2016). This is consistent with our previous placement of A297/RUH875 in the ST1 phylogenetic tree.^20^

### Conclusions

The complete genome sequence of the multiresistant RUH875 (A297) provides better insights into its genomic features, including antibiotic resistance regions, plasmids and its distinct lineage within GC1. As a reference GC1 strain and one of the earliest strains available, completing its genome is crucial for future AMR studies of the globally distributed clone 1. Its detailed characterization advances our understanding of AMR evolution in early GC1 isolates and facilitates future investigations into the genetic diversity and adaptation of this globally distributed clone.
